# Impaired ossification coupled with accelerated cartilage degeneration in developmental dysplasia of the hip: evidences from μCT arthrography in a rat model

**DOI:** 10.1186/1471-2474-15-339

**Published:** 2014-10-08

**Authors:** Ming Fu, Jin Liu, Guangxin Huang, Zhiyu Huang, Zhiqi Zhang, Peihui Wu, Bingjun Wang, Zibo Yang, Weiming Liao

**Affiliations:** Department of Joint Surgery, First Affiliated Hospital of Sun Yat-sen University, No. 58 Zhongshan 2nd road, Guangzhou, 510080 China; Institute for Advancing Translational Medicine in Bone & Joint Disease, School of Chinese Medicine, Hong Kong Baptist University, Hong Kong, SAR China

**Keywords:** Developmental dysplasia of the hip, μCT arthrography, Impaired ossification, Cartilage degeneration

## Abstract

**Background:**

Developmental dysplasia of the hip (DDH) always leads to cartilage degeneration and osteoarthritis of the hip joint. However, the diagnosis of early cartilage degeneration in DDH is still a clinical challenge. This study aims to investigate the dynamic changes of bone and cartilage in the hip of a rat model of DDH and to explore the potential application of microcomputed tomography (μCT) arthrography to detect early cartilage degeneration in DDH.

**Methods:**

Newborn Wistar rats were used to induce DDH by hindlimb swaddling. The bone and cartilage of the hip in model and control group were analyzed by μCT arthrography and histology examination at postnatal day 10, week 4, 6 and 8.

**Results:**

Hip dysplasia developed with age, became obvious at postnatal week 6 and further progressed at week 8. μCT analysis showed that bone mineral density (BMD) and bone volume density (bone volume over total volume, BV/TV) of the femoral head and neck region (FHNR) in model group were both significantly lower than those in control group, and they increased dramatically from postnatal week 4 to week 6 but maintained at a similar level at week 8. Contrast-enhanced μCT (CE-μCT) arthrography and histology data showed age-dependent increase in cartilage attenuation (CA) and decrease in safranin O staining intensity (SI) in model group, respectively. Moreover, the model group revealed remarkably higher CA and lower SI than control group, respectively. In addition, significant changes of CA and SI were both observed from postnatal week 6 to week 8 in model group. A strong linear correlation (r^2^ = 0.789, P <0.001) was found between CA and SI in model group. Furthermore, BMD was negatively correlated with SI (t = -2.683, P <0.05), whereas specific bone surface (bone surface over bone volume, BS/BV) was positively correlated with SI (t =4.501, P <0.01), in model group.

**Conclusions:**

Impaired ossification coupled with continuous loss of sGAG in cartilage matrix was found in the dysplasia hip during the disease progression of DDH. Cartilage degeneration in the dysplasia hip may occur early at childhood, accelerated with age and become irreversible at young adult stage. All these abnormal changes could be quantitatively assessed by μCT arthrography.

**Electronic supplementary material:**

The online version of this article (doi:10.1186/1471-2474-15-339) contains supplementary material, which is available to authorized users.

## Background

Developmental dysplasia of the hip (DDH) is a common developmental skeleton defect [1]. Although screening and management for DDH has been developing during the decades [2], the number of late cases is not decreasing due to its insidious onset [3]. A part of patients who received improper treatment in childhood may develop into residual DDH. For these patients, most of them would progress to severe hip osteoarthritis (OA) which requires total hip arthroplasty (THA), if cartilage degeneration of the dysplasia hip could not be detected early and treated appropriately. However, the surgery is more complicated and the incidence of postoperative complications is much higher when comparing to other primary THAs [4]. Therefore, the best strategy to prevent the dysplasia hip into sever OA is to diagnose it as early as possible and to reestablish the normal contact between femoral head and acetabulum before the onset of irreversible degeneration of the hip cartilage [5]. Nevertheless, the unconspicuous symptoms and signs of the early-stage cartilage degeneration in DDH patients make early diagnosis difficult. The key issues are when such degenerative process begins and when it becomes irreversible in DDH, and more importantly, how to detect it sensitively and early. At present, there is still lack of knowledge regarding to these questions.

Previous studies have well documented the natural history of DDH, in which the deformity of hip bone contributes to aberrant stress on the joint cartilage [6]. Moreover, the loss of sulphated glycosaminoglycan (sGAG) in cartilage matrix was considered to be characteristic for early cartilage degeneration [7]. Thus, we conducted this study in a rat model of DDH to investigate the pattern of bone development as well as the dynamic changes of sGAG content in dysplasia hip during the disease progression of DDH by μCT arthrography and histology examination. μCT imaging can provide nondestructive three-dimensional (3-D) analysis of the bone [8, 9]. Contrast-enhaced μCT (CEμCT) can quantitatively reflect sGAG content in the cartilage matrix by measuring the equilibrium partitioning of the ionic contrast agent Hexabrix 320 due to the loss of negatively charged sGAG in cartilage matrix [10]. Results of μCT arthrography will be compared with histological findings and the correlation between them will be further tested. We hypothesized that the abnormal biomechanical condition of DDH impairs postnatal development of hip bone and cartilage, and it further induces early degeneration of joint cartilage, which could be quantitatively assessed by μCT arthrography.

## Methods

### Animals and DDH model

Six pregnant Wistar rats were used in the study. The pregnant rats were housed in dark and quiet environment and the gestational period ranged from 21 to 23 days. A total of 32 female newborn rats were randomly selected and divided into model group (n =20) and control group (n =12) and breastfed by their mothers till postnatal day 28. Thereafter, they were kept in separate cages and fed with standard diet. All the rats were maintained under standard animal housing conditions (12-h light, 12-h dark cycles and free access to food and water) in the Laboratory Animal Center of Sun Yat-sen University. In model group, the rats’ hindlimbs were fixed together at the position of hip adduction and extension as well as knee extension with medical tapes for the first postnatal 10 days as previously reported [11, 12]. The straight-leg swaddling method was performed in a manner that simulated human swaddles (Additional file 1: Figure S1). During the first 10 days, the rats were released from the swaddling and allowed unrestricted motion for an hour per day. After 10 days, the swaddles were removed. In control group, there was not any intervention on the rats. Sequentially, five rats from model group and three rats from control group were randomly chosen to be sacrificed at postnatal day 10, week 4, 6 and 8. The rationale behind these 4 time points was that the time points corresponds to human infancy stage, child stage, teenager stage and young adult stage, respectively. In different ages, necropsy specimens of the whole pelvis with bilateral hip joints were isolated from rats in both groups after euthanasia. All the procedures for animal models were approved by the Sun Yat-sen University ethics committee (Approval No. IACUC-2012 0704).

### Sample management and macroscopic observation

At different ages above, the pelvises with bilateral hips were harvested after the rats were killed. The muscle around the hip joint was dissected carefully, reserving the joint capsule. Thereafter, the necropsy specimens were photographed and then kept in phosphate buffered saline (PBS) with protease inhibitor (Complete, EDTA-free Protease Inhibitor Cocktail Tablets, Roche) at 4°C till μCT scan [13]. Several punctures were made in the hip capsule with 21 gauge needle to allow the penetration of the preserving reagent. Each sample was scanned by μCT for twice. And immediately after the first time μCT scan, the hip capsule was removed and the femoral head were separated from the acetabulum, photos of the femoral head and acetabulum were subsequently captured and the specimens were subjected to CEμCT scan.

### μCT scan and analysis

The whole pelvises with bilateral hips from both groups at each time point were scanned ex vivo using a μCT system (ZKKS-MCT-Sharp, Zhongke Kaisheng Medical Technology, Guangzhou, China) at 60kVp and 667 μA. Briefly, 250 slices with a voxel size of 20 μm were acquired in the region including the entire pelvis, the hip joint and the proximal 1/3 of the femur. All the slices were segmented by the built-in software (3DMed 4.1, Zhongke Kaisheng Medical Technology, Guangzhou, China) to provide a 3-D view of the entire bony structure of the pelvis with bilateral hips. Thereafter, 80 slices including the femoral head and neck region (FHNR) on the XY plane (the anteroposterior view) were selected and segmented for 3-D reconstruction of the bony structure of this region with the following parameters calculated: BMD (bone mineral density), BV/TV (bone volume over total volume, bone volume density), BS/BV (bone surface over bone volume, specific bone surface).

### Assessment of acetabular angle

The μCT slices on the XY plane revealed the entire pelvis in an anteroposterior (coronal) view. To determine the radiographic acetabular angle (AA), the slice showing the widest inferior margin of ilium was selected, then one line (line A) was drawn between the intersection point of the ilium and ischium at each side and another line (line B) was drawn across the outer edge of the inferior margin of ilium and the above-mentioned intersection point (Additional file 2: Figure S2). Accordingly, the AA was defined as the angle formed by the two lines and was calculated for each specimen.

### CE-μCT scan and analysis

After the first μCT scan, the specimens were taken out from the scan cabin and joint capsule was incised to separate the femoral head from acetabulum. The entire surface of femoral head cartilage was then exposed and immersed in contrast medium (40% Hexabrix 320 and 60% PBS) and water bathed at 37°C for 30 minutes [13]. Thereafter, the specimens were gently patted dry and immediately placed on the fixture and scanned by the same μCT system at 60kVp and 667 μA. The total slices with the entire FHNR including cartilage, subchondral bone and trabecular bone were identified on the XY plane images. A histogram of the X-ray attenuation values was first produced by the built-in software, revealing two peaks corresponding to contrast-enhanced articular cartilage and calcified bone. To distinguish the cartilage from the bone, the selected slices were segmented by manually assigning the lower and upper thresholds to isolate the voxels within the cartilage peak, which unavoidably contained a few bone marrow voxels. To determine the cartilage attenuation (CA) of the femoral head cartilage layer, contour lines were manually drawn on every selected slice to eliminate marrow space and the average attenuation was calculated with all the region of interest (ROI) within the contour lines in the selected slices. In addition, the distribution of contrast reagent in femoral head cartilage layer was displayed by pseudo-color in the medial slice of each specimen.

### Histological analysis

Following μCT scan, all femoral head specimens were fixed in 4% paraformaldehyde for 24 hours, decalcified in 10% EDTA for 3 to 4 weeks. Dehydrated specimens were embedded in paraffin and coronal sections were sequentially cut at 5 μm thickness. Sections were deparaffinized in xylene and graded ethanol, stained for sGAGs with a 0.5% safranin-O solution and a 0.2% aqueous solution of fast green as a counterstain. Thereafter, the stained sections were dehydrated, cleared, mounted with cover slip. Sections containing the medial area of the femoral head cartilage were selected and digital images were captured with the Olympus BX51 optical microscope. The images were then exported to Image-pro Plus 6.0 software, and the ROI of cartilage layer was selected to calculate the safranin-O SI of this region to reflect the content of sGAG.

### Statistical analysis

All quantitative data in this study were presented as mean values with standard deviations. Data were analyzed using the SPSS 19.0 statistical software (IBM SPSS Statistic 19.0). Two-way analysis of variance (ANOVA) and Turkey’s test for post-hoc analyses were used for comparison between the two groups across different time points. The multiple-linear regression model was used to evaluate the correlation between μCT parameters of bony structure and SI. The general-linear regression model was used to analyze the relationship between CA and SI with all the data from model group pooled together. Statistical significance was set at *P* <0.05.

## Result

### Rats in model group showed morphological deformity of the dysplasia hips

**Figure 1:**
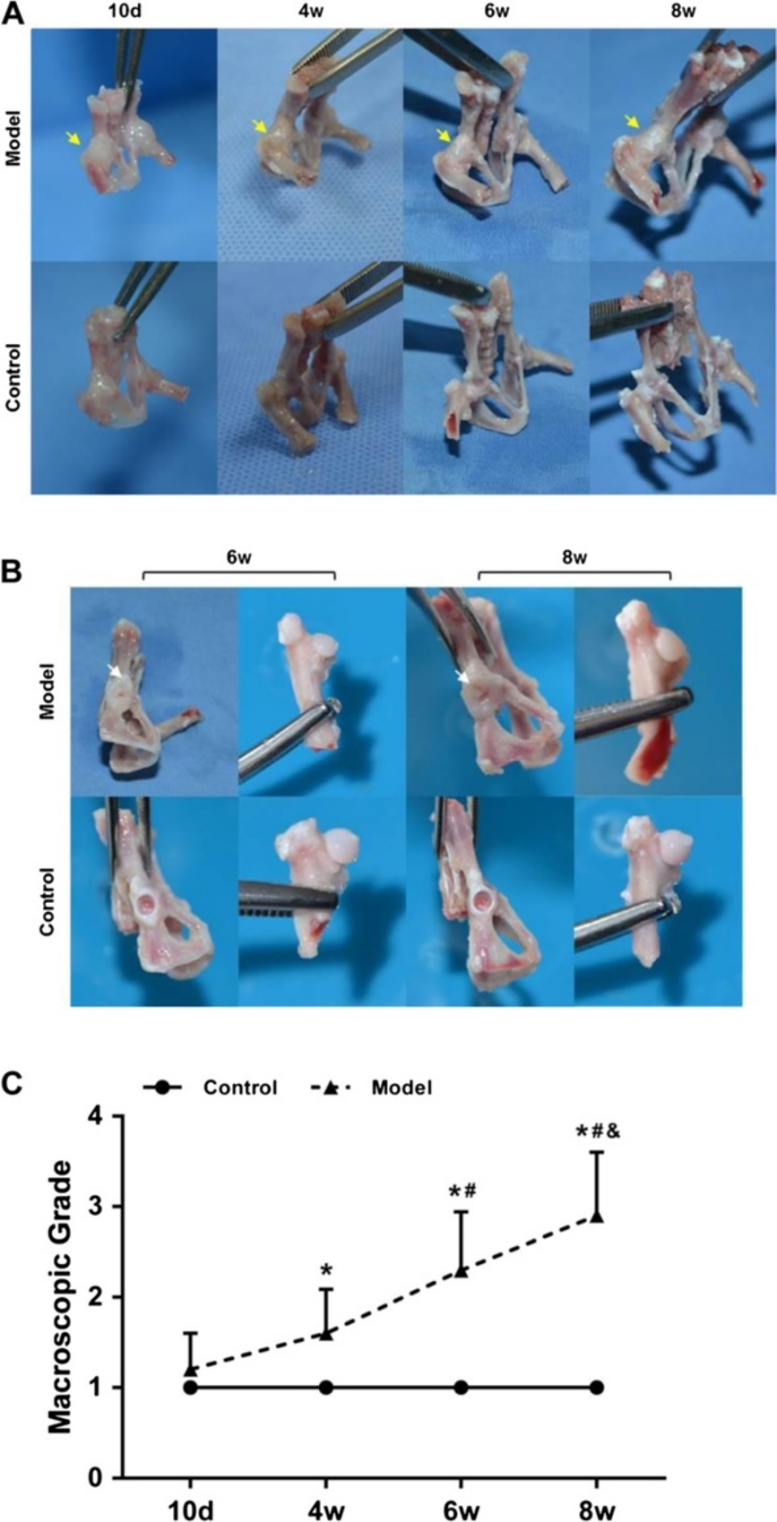
**Time course changes of the macroscopic features of the hips. (A)** General view of the hips in each group at different time points (yellow arrows indicating the thickening of the joint capsule). **(B)** Local appearances of the acetabulum and the femoral head in each group at the end of post-natal week 6 and week 8 (white arrows indicating the false acetabulum). **(C)** Macroscopic grade of the femoral head cartilage in each group at different time points. Note: *: *P* <0.05 vs mice in control group; #: *P* <0.05 vs mice at post-natal day 10*;* &: *P* <0.05 vs mice at post-natal week 4*.*

**Figure 2:**
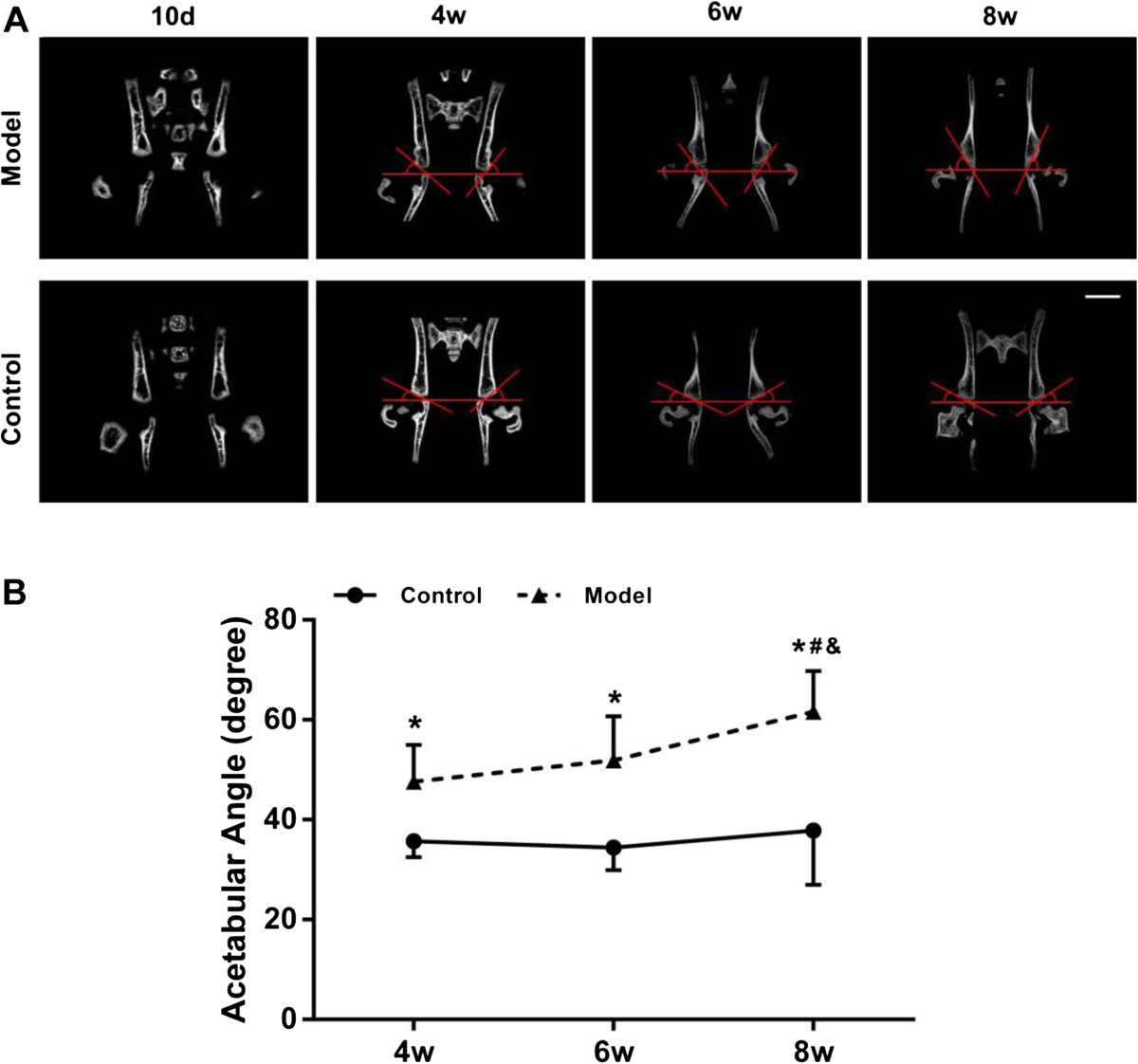
**Time course changes of the acetabular angles (AA). (A)** Representative anterior-posterior views of the AA in each group at different time points by two-dimensional μCT imaging. **(B)** Quantitative analysis of the AA in each group at different time points. Note: *: *P* <0.05 vs mice in control group; #: *P* <0.05 vs mice at post-natal week 4*;* &: *P* <0.05 vs mice at post-natal week 6*.*

**Figure 3:**
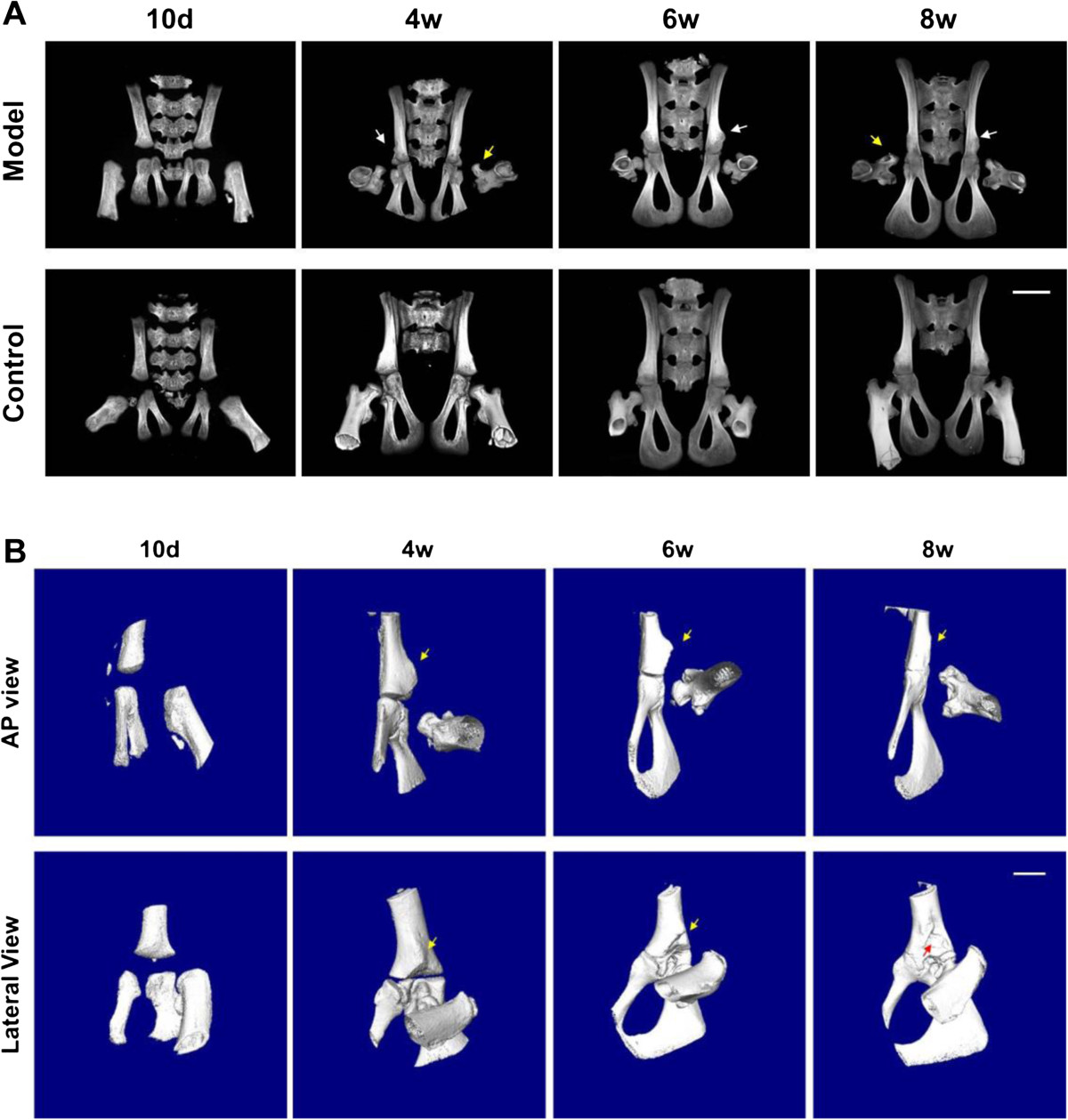
**Time course changes of the hip bony structure by μCT based three-dimensional reconstruction. (A)** General views of the hip bony structure in each groupat different time points. **(B)** The hip bony structure in model group. Note: the white arrows indicating shallowing of the acetabulum; the yellow arrows indicating malposition of the femoral head; the red arrows indicating false acetabulum.

No obvious differences in body weight and length were found between the rats in model and control groups from postnatal day 10 to week 8 (data not shown). Thickening of the hip joint capsule was found in model group at each time point. In contrast, the joint capsule in control group was thin and transparent enough for the visualization of joint cartilage (Figure 1A). Shallow acetabulum and irregular flat femoral head was also found in model group and false acetabulum was further observed in some of the specimens at postnatal week 6 and week 8 (Figure 1B). The macroscopic grades of the hip cartilage in model group increased dramatically from postnatal day 10 to week 8, whereas they showed minimal changes in control group (Figure 1C).Because of the cartilaginous structure in hip in the early postnatal period, the hip containment and AA in both groups could not be measured in μCT arthrography until postnatal week 4. The outer edge of acetabulum in model group was too steep to offer enough coverage to femoral head (Figure 2A). Quantitatively, the average degree of AA in model group was 47.6 ± 7.3 at postnatal week 4, increased slightly to 51.9 ± 8.8 at postnatal week 6 and raised dramatically to 61.6 ± 8.1 at postnatal week 8 (P <0.05), while the average AA in control group maintained at a low degree. Furthermore, AA in model group was significantly higher (33.6%, 50.8% and 63.1%, P <0.01, respectively) than control group at the later 3 time points (Figure 2B). According to μCT reconstruction images, hip deformity in model group was more distinguished with increasingly shallow acetabulum and malposition of the femoral head from postnatal week 4 to week 8 (Figure 3). Notably, false acetabulums in some of the specimens from model group at week 6 and week 8 were also imaged by μCT (Figure 3B).

### Advanced but impaired ossification of the femoral head and neck region was found in the dysplasia hips

**Figure 4:**
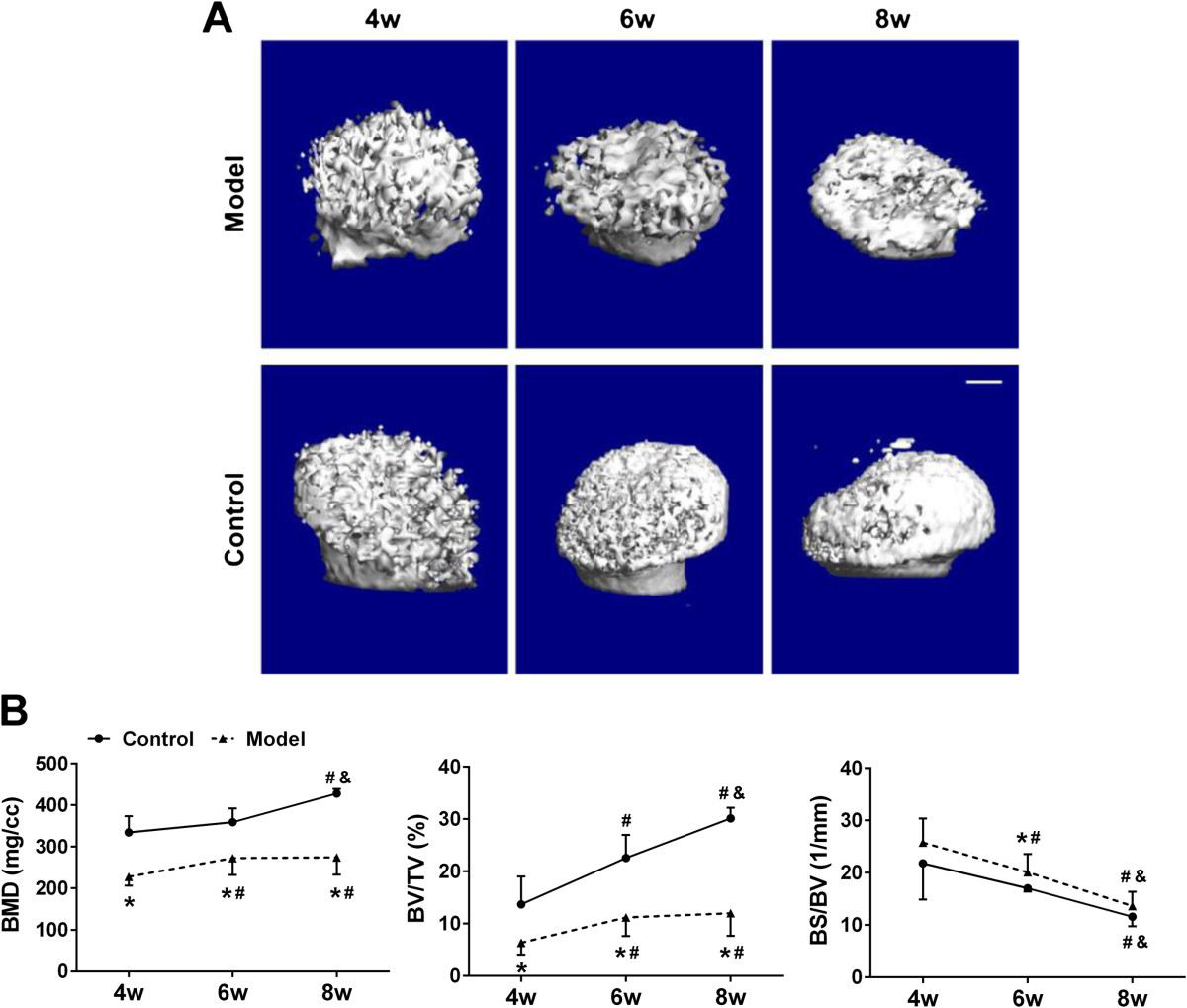
**Time course changes of the bony structure of the femoral head and neck region by three-dimensional μCT analysis. (A)** Representative three-dimensional μCT reconstructed images of the femoral head and neck region in each group at post-natal week 4, 6 and 8. **(B)** Quantitative analysis of the bony structure of the femoral head and neck region in each group at post-natal week 4, 6 and 8. Note: *: *P* <0.05 vs mice in control group; #: *P* <0.05 vs mice at post-natal week 4*;* &: *P* <0.05 vs mice at post-natal week 6*.*

As shown in 3-D μCT images, the FHNR in model group appeared flat and irregular shapes without significant changes in size with age, whereas those in control group showed steady enlargement of the size and reformation of a hemisphere-like femoral head (Figure 4A). BMD and BV/TV increased while BS/BV decreased with age in both groups. As compared to control group, BMD and BV/TV in model group were significantly lower (P <0.01) at the later 3 time points, whereas BS/BV were remarkably higher (P <0.05) only at postnatal week 6 (Figure 4B). Interestingly, BMD and BV/TV in model group increased dramatically from postnatal week 4 to week 6 (19.9% and 75.6%, P <0.05, respectively) but maintained at a similar level from week 6 to week 8 (0.5% and 7.4%, respectively). However, the two parameters in control group increased steadily across the time points (Figure 4B).

### Impaired development and accelerated degeneration of the joint cartilage was found in the dysplasia hips

**Figure 5:**
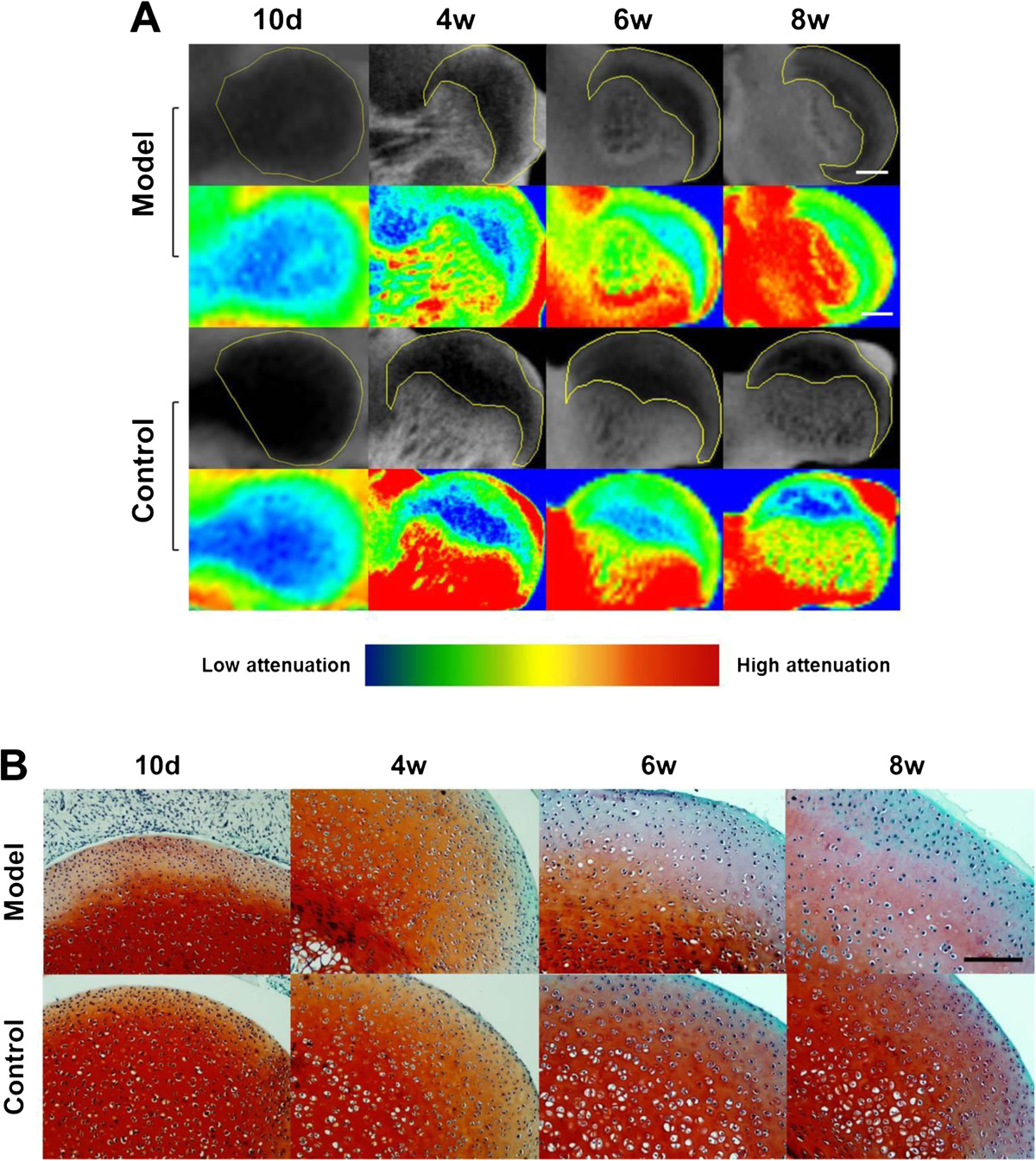
**Representative CE-μCT images (A) and corresponding histological sections (B) of the femoral head cartilage in each group at different time points.**

**Figure 6:**
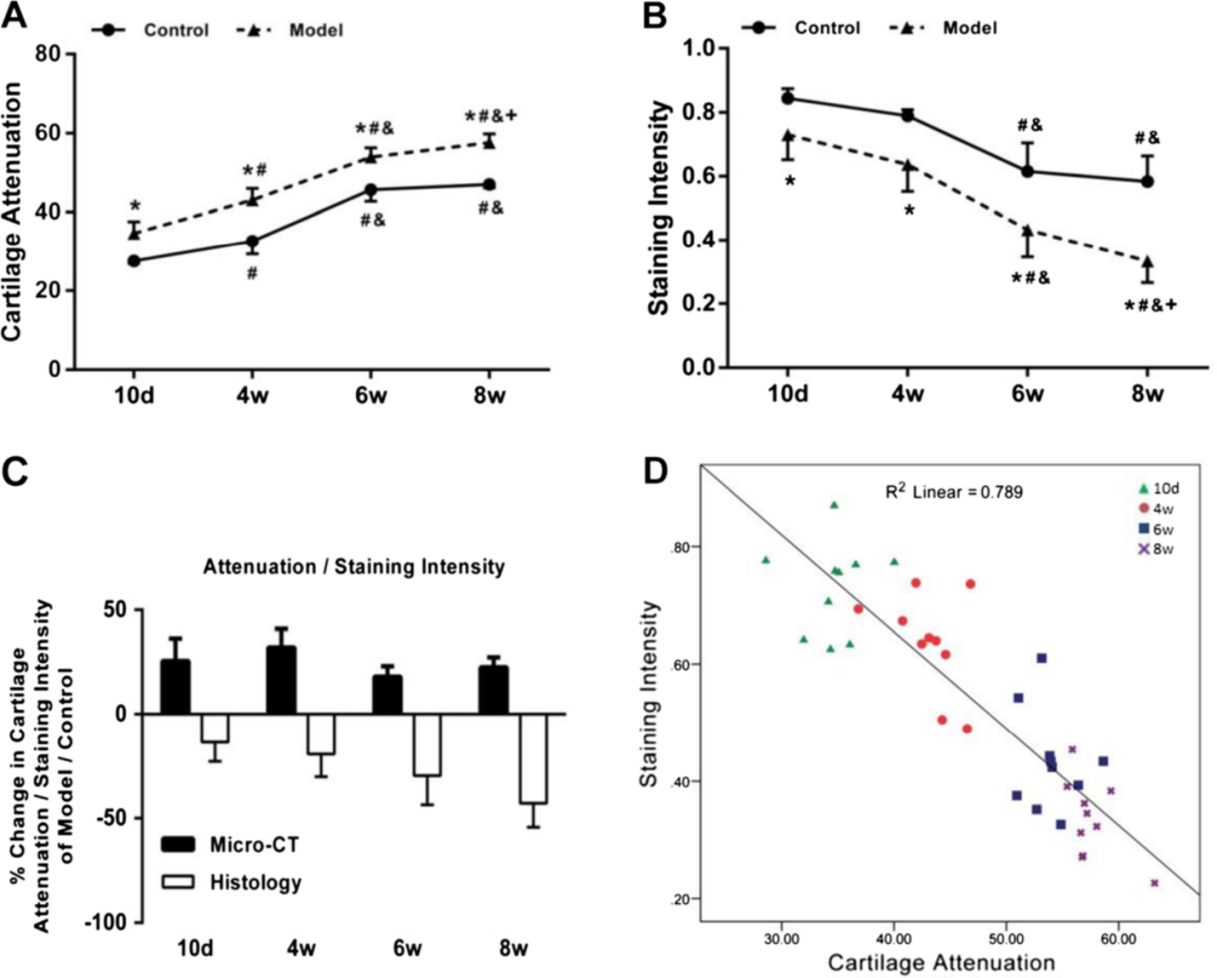
**Quantitative analysis of the changes in cartilage attenuation of CE-μCT images and staining intensity of the safranin-O stained histological sections. (A)** Time course changes of the average cartilage attenuation of the femoral head cartilage layer in each group. **(B)** Time course changes of the average staining intensity of the femoral head cartilage layer in each group. **(C)** Percentage cartilage attenuation and staining intensity changes of femoral head cartilage layer from the model group over the control group. **(D)** Linear regression of cartilage attenuation vs staining intensity for femoral head specimens pooled across all the time points in model group. Note: *: *P* <0.05 vs mice in control group; #: *P* <0.05 vs mice at post-natal day 10*;* &: *P* <0.05 vs mice at post-natal week 4*;* +: *P* <0.05 vs mice at post-natal week 6.

According to the CE-μCT analysis, CA in femoral head cartilage layer increased with age, while according to the safranin-O stained histology examination, SI of the corresponding cartilage area decreased with age (Figure 5). The results may attribute to endochondral ossification in femoral head during postnatal development. On the other hand, CA was obviously higher, while SI was obviously lower, in model group than those in control group, respectively, indicating enhanced loss of the sGAG in joint cartilage of dysplasia hip (Figure 5). Consistent with the visualized estimation, quantitative analysis further proved that CA in model group was 25.6%, 32%, 18% and 22.5% higher than control group at postnatal day 10 and week 4, 6 and 8, respectively, while SI in model group was 13.5%, 19.3%, 29.6% and 42.8% lower than control group at the corresponding time point, respectively (Figure 6A-C). All the differences above between the two groups were statistically significant (P <0.01). Notably, the age-dependent increase in CA and decrease in SI both peaked at postnatal week 8 in model group (P <0.05), whereas remarkable increase in CA and decrease in SI was only detected from postnatal week 4 to week 6 in control group (P <0.05), respectively (Figure 6A & B).

### μCT arthrography could predict the extent of sGAG loss in joint cartilage of the dysplasia hips

In addition, a strong linear correlation (r^2^ = 0.789, slope = -0.17, P <0.001, n =20; Figure 6D) between CA and the SI was detected after analysis of the femoral head specimens pooled across ages in model group. Moreover, multiple linear regression analysis showed that BMD was negatively correlated (t = -2.683; P <0.05), while BS/BV was positively associated (t =4.501; P <0.01) with SI (Table 1).

**Table 1:** **Multiple linear regression of three-dimensional μCT parameters of the bony structure of the femoral head and neck regions and the staining intensity of the safranin-O stained femoral head section**

Parameters	Beta	t	*P.*
BMD	- 0.553	- 2.638	0.014*
BV/TV	0.38	1.429	0.165
BS/BV	0.795	4.501	0.000*

*: *P* <0.01.

## Discussion

In the present study, we built a hindlimb swaddling model to induce DDH in newborn Wistar rats as previously reported [11, 12] and to investigate the dynamic changes of bone and cartilage during postnatal development in dyaplasia hips. We applied μCT arthrography to evaluate bone and cartilage quality in dysplasia hips by comparison with histological findings. Dysplasia hip was successfully induced, which was observed immediately after the swaddling was removed, developed with age, became obvious at postnatal week 6 and further progressed at week 8. The ossification of the FHNR was advanced but impaired in dysplasia hips. The extent of sGAG loss in joint cartilage was much more significant in dysplasia hips than that in normal controls. Furthermore, BMD and BS/BV calculated by μCT and cartilage CA measured by CE-μCT were found to be sensitive predictors for the content of sGAG in femoral head cartilage in dysplasia hips.

According to the macroscopic results, we observed notable morphological changes of the bony structure of the dysplasia hip in model group including the shallow acetabulum and flat femoral head, which are in concordance with the findings of other studies [11, 12]. The average degree of AA was significantly higher in model group than control group and increased remarkably from postnatal week 6 to week 8. Moreover, the formation of false acetabulum was also detectable at the later time points. These results implied the deformity of bony structure in dysplasia hip aggravated from postnatal week 6 to week 8. On the other hand, we also detected significantly lower BMD and BV/TV in the FHNR of model group when compared to control group by μCT analysis. It has been reported that BMD at the hip in adult DDH patients who received conservative treatment in childhood was significantly lower than normal controls [14], whereas another study found that BMD at calcaneus of proximal femur was significantly higher in patients with severe DDH (average central-edge angle was 6.7°) than in controls [15]. Thus, the relationship between BMD and DDH is still controversial, which may attribute to the different stages of DDH as well as different treatments the patients received. Additionally, BMD was also identified as a predictor for osteoarthritis in several animal studies [16, 17]. Furthermore, BMD is an accepted parameter in determining the status of bone mineralization [18]. Considering that the rats used in this study were so young that the hips were still under postnatal development characterized by endochondral ossification, it is reasonable to find the FHNR of DDH rats with lower BMD than normal controls as a result of impaired endochondal ossification. On the other hand, we also observed different tendencies in the dynamic changes of BMD and BV/TV of FHNR between model and control groups. Interestingly, the two parameters peaked at postnatal week 6 in model group, whereas they continued to increase from postnatal week 6 to week 8 in control group. This result probably suggested an earlier termination of the endochondal ossification of the femoral head cartilage in DDH subjects, which further proved the impaired ossification of dysplasia hip during postnatal development and might explained the bone deformity of the hip observed in DDH rats at different stages.

Our histological data demonstrated significant lower content of sGAG in the cartilage matrix of dysplasia hip than normal controls at different postnatal stages, indicating the impairment of cartilage development during the disease progression of DDH. We also found age-dependently increasing loss of sGAG in femoral head cartilage of DDH rats, implying the early onset of cartilage degeneration at childhood and the remarkable progression of the degenerative changes in dysplasia hips with age. It should be noted that endochondral ossification is another postnatal factor leading to the loss of sGAG in the femoral head cartilage [19, 20]. During this normal developmental process, the content of sGAG decreased mildly as evidenced by the slightly decrease of safranin-O SI with age in control group by histology analysis. Further, remarkable decrease of SI was found only from postnatal week 4 to week 6 in control group, indicating the peak of endonchondral ossification in this period. However, significant decrease of SI in model group was observed from postnatal week 4 to week 6 and continued to week 8, suggesting an extensive loss of sGAG in the cartilage matrix of dysplasia hip at this later time point. Our results were consistent with a previous study showing that degenerative cartilage changes occurred in a similar DDH model at postnatal week 4 and became aggravated with age [12]. In that study, Bo and colleagues observed continuous loss of sGAG and significantly increased expression of type X collagen and MMP-13, two markers of cartilage degeneration, in dysplasia hip as compared to controls. Moreover, the mRNA level of MMP-13 was dramatically elevated from postnatal week 6 to week 8. Given the knowledge that MMP-13 mainly contributes to the degeneration of type II collagen during cartilage degeneration [21, 22], high expression of this collagenase is related with extensive degeneration of type II collagen [22], which has been recognized as irreversible changes for cartilage degeneration [23]. Taken together, it suggests the irreversible cartilage degeneration may start from postnatal week 6 to week 8 in DDH rats. Additionally, the time point were also consistent with the time when AA increased significantly and false acetabulum formed, indicating that the remarkable bone deformity may be another signal for the irreversible cartilage degeneration in dysplasia hip.

Currently in clinical practice, it is impossible to performed hip cartilage biopsy on patients with DDH at different stages to diagnose cartilage degeneration. One alternative but also the most applicable approach is to use imaging technique to diagnose these patients and to detect signs for cartilage degeneration. Though several studies have demonstrated the application of radiography, CT and MRI to diagnose the DDH-related cartilage degeneration and secondary OA in patients [24–26], there is still a lack of research to directly compare the histology results of cartilage degenerative changes with the corresponding imaging finding in dysplasia hip. In this study, we found that BMD/BS/BV was negatively/positively associated with the content of sGAG in femoral head cartilage of DDH rats, indicating that the two parameters might be promising predictors to reveal the level of early cartilage degeneration in DDH. However, the age-dependent change of BS/BV was similar to the pattern of the decreasing SI in model group, and the correlation between them are much stronger as evidenced by a much smaller p-value for BS/BV than BMD in the multiply linear regression analysis. Collectively, BMD and BS/BV might be potential predictors for the content of sGAG in dysplasia hip and the latter may have higher consistency with the decrease of sGAG in hip cartilage during the disease progression of DDH.

On the other hand, we also verified that femoral head cartilage of DDH rats could be imaged by CE-μCT. The dynamic change of CA was opposite to the alteration of safanin-O SI, that is, higher CA paralleled to lower sGAG content and vice versa. Thus, by calculating CA, the content of sGAG could be measured in dysplasia hip. As we know, CE-μCT was first introduced by Palmer and colleagues as a novel nondestructive method to quantitative assessement of cartilage composition and morphology [10]. A series of pilot studies have already evidenced the successfully application of this imaging technique, both ex vivo [10, 13, 27–29] and in vivo [30], to reveal the stage of degraded cartilage by quantitatively evaluating the distribution and content of sGAG in murine and rabbit models with knee OA. Further, a similar technique by clinical CT scan has been proved to be sensitive to assess cartilage quality on human cadaveric knee joints in a couple of recent pre-clinical researches [31, 32]. However, published studies on the application of CE-μCT to measure the sGAG content all focused on the knee joint instead of the hip, which we think may attribute to the superficial location of the knee joint and the well-established protocols to induce OA-like changes in the cartilage of the knee in animal models. It should be aware that delayed gadolinium-enhanced MRI of cartilage (dGEMRIC), another novel technique similarly designed to examine sGAG changes in articular cartilage, has been demonstrated to be capable of predicting the degree of cartilage degeneration of the dysplasia hip in DDH patients [33–35]. However, all these clinical studies shared the same limitations as mentioned above that they failed to bridge the imaging findings with in situ histological changes of the degraded cartilage. Considering that the two imaging techniques shared similar mechanism for quantitative analysis the sGAG content in cartilage matrix, our study provided histological evidences to the extent of sGAG loss in the dysplasia hip and further established the correlation between CE-μCT and histology examination.

To the best of our knowledge, this is the first study to apply μCT arthrography to evaluate the age-dependent changes of bone and cartilage in dysplasia hip during the postnatal progression of DDH, and we preliminarily bridge the imaging parameters with histological findings in cartilage degeneration of the dysplasia hip. It may provide another powerful and sensitive approach for research and diagnose of DDH. However, there were several aspects in this study, including the relatively short observation period and the ex vivo μCT scan owning to the difficulty in intra-articular injection of the contrast reagent to the hip, which limited a more comprehensive understanding of the issue discussed here. It will be improved in our extended study in future.

## Conclusions

We have found impaired ossification coupled with continues loss of sGAG in cartilage matrix of the dysplasia hip during the disease progression of DDH. Cartilage degeneration in the dysplasia hip occurs early at childhood, aggregates with age and becomes irreversible at young adult stage. All these abnormal changes could be quantitatively assessed by μCT arthrography.

## Electronic supplementary material

Additional file 1: Figure S1: Details of the DDH (straight-leg swaddling) model. (A) Representative images showing the methods to establish straight-leg swaddlingmodel. (B) Representative images showing the appearances of the rats in model and control groups at postnatal 5 days and 10 days, respectively. (C) The body length and weight of the rats in model and control group, respectively. (PDF 131 KB)

Additional file 2: Figure S2: The μCT slice on the XY plane showing the widest inferior margin of ilium was selected. Line A (red) was drawn between the intersection point of the ilium and ischium at each side and line B (yellow) was drawn across the outer edge of the inferior margin of ilium and the above-mentioned intersection point. Acetabular angle (AA) was defined as the angle formed by the two lines (blue). (PDF 111 KB)

Below are the links to the authors’ original submitted files for images.

Authors’ original file for figure 1

Authors’ original file for figure 2

Authors’ original file for figure 3

Authors’ original file for figure 4

Authors’ original file for figure 5

Authors’ original file for figure 6

## Abbreviations

DDH: Developmental dysplasia of hip
OA: Osteoarthritis
THA: Total hip arthroplasty
μCT: Micro-computed tomography
CE-μCT: Contrast-enhanced micro-computed tomography
FHNR: Femoral head and neck region
BMD: Bone mineral density
BV/TV: Bone volume over tissue volume
BS/BV: Bone surface over bone volume
AA: Acetabular angle
CA: Cartilage attenuation
SI: (safranin O) staining intensity
ANOVA: Analysis of variance
sGAG: Sulphated glycosaminoglycan
MRI: Magnetic resonance image
dGEMRIC: Delayed gadolinium-enhanced MRI of cartilage.
