# Supplementary material for: Impaired ossification coupled with accelerated cartilage degeneration in developmental dysplasia of the hip: evidences from μCT arthrography in a rat model
Source: BMC Musculoskelet Disord. 2014 Oct 8;15:339. doi: 10.1186/1471-2474-15-339 (PMC4289046; doi:10.1186/1471-2474-15-339)
Supplement: Supplementary file 1 — Additional file 1: Figure S1: Details of the DDH (straight-leg swaddling) model. (A) Representative images showing the methods to establish straight-leg swaddlingmodel. (B) Representative images showing the appearances of the rats in model and control groups at postnatal 5 days and 10 days, respectively. (C) The body length and weight of the rats in model and control group, respectively. (PDF 131 KB) [file 12891_2014_2374_MOESM1_ESM.pdf]

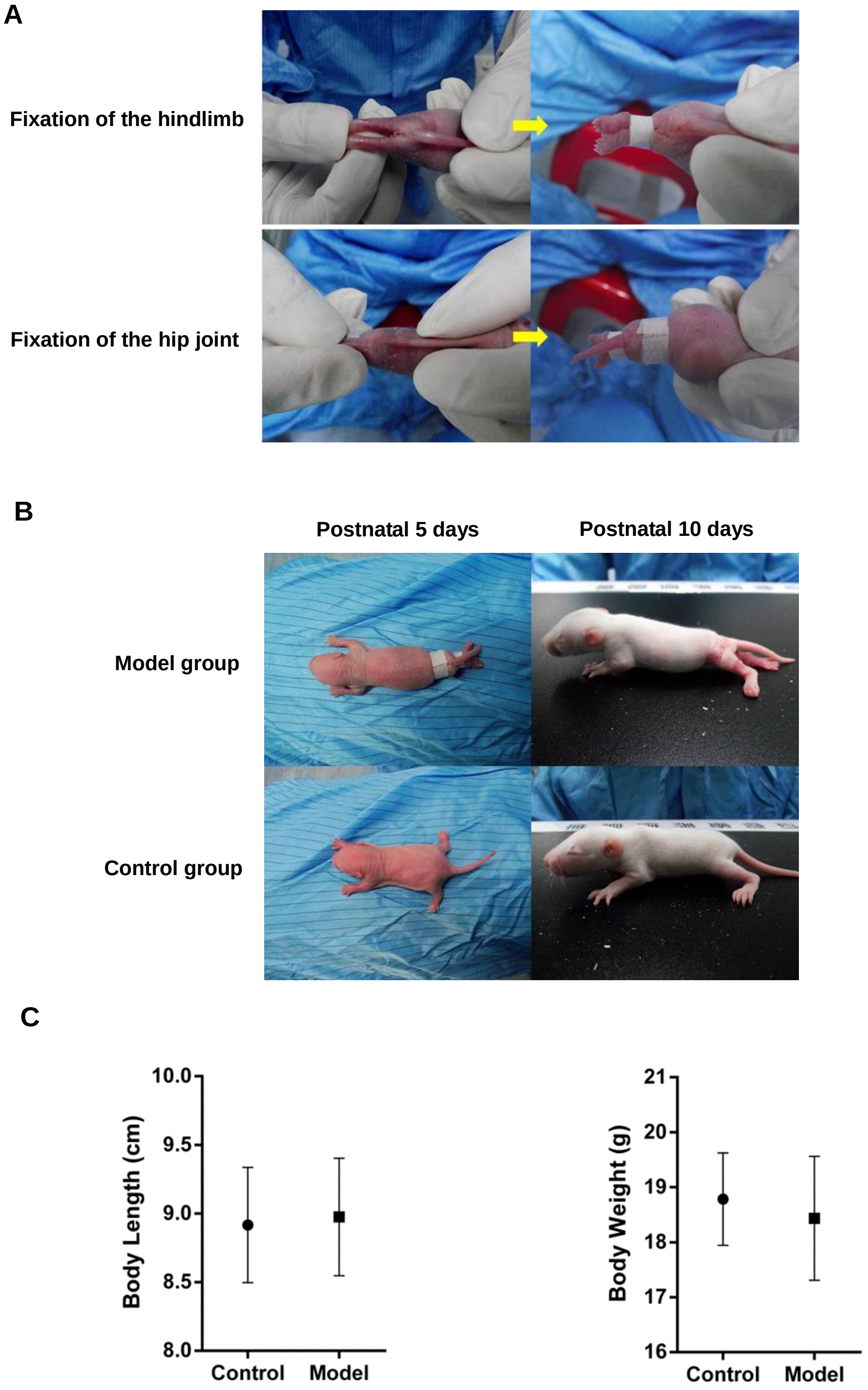

Figure 1 Details of the DDH (straight-leg swaddling) model (A) Representative images showing the methods to establish straight-leg swaddling model. (B) Representative images showing the appearances of the rats in model and control groups at postnatal 5 days and 10 days, respectively. (C)The body length and weight of the rats in model and control group, respectively.
