# Supplementary material for: Impaired ossification coupled with accelerated cartilage degeneration in developmental dysplasia of the hip: evidences from μCT arthrography in a rat model
Source: BMC Musculoskelet Disord. 2014 Oct 8;15:339. doi: 10.1186/1471-2474-15-339 (PMC4289046; doi:10.1186/1471-2474-15-339)
Supplement: Supplementary file 2 — Additional file 2: Figure S2: The μCT slice on the XY plane showing the widest inferior margin of ilium was selected. Line A (red) was drawn between the intersection point of the ilium and ischium at each side and line B (yellow) was drawn across the outer edge of the inferior margin of ilium and the above-mentioned intersection point. Acetabular angle (AA) was defined as the angle formed by the two lines (blue). (PDF 111 KB) [file 12891_2014_2374_MOESM2_ESM.pdf]

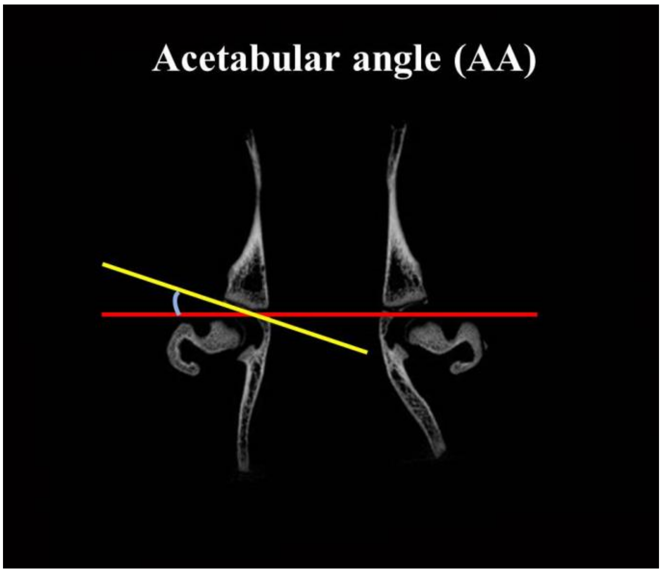


**Supplementary Figure 2** The μCT slice on the XY plane showing the widest inferior margin of ilium was selected. Line A (red) was drawn between the intersection point of the ilium and ischium at each side and line B (yellow) was drawn across the outer edge of the inferior margin of ilium and the above-mentioned intersection point. Acetabular angle (AA) was defined as the angle formed by the two lines (blue).
