# Supplementary figures and images for: Impaired ossification coupled with accelerated cartilage degeneration in developmental dysplasia of the hip: evidences from μCT arthrography in a rat model
Source: BMC Musculoskelet Disord. 2014 Oct 8;15:339. doi: 10.1186/1471-2474-15-339 (PMC4289046; doi:10.1186/1471-2474-15-339)

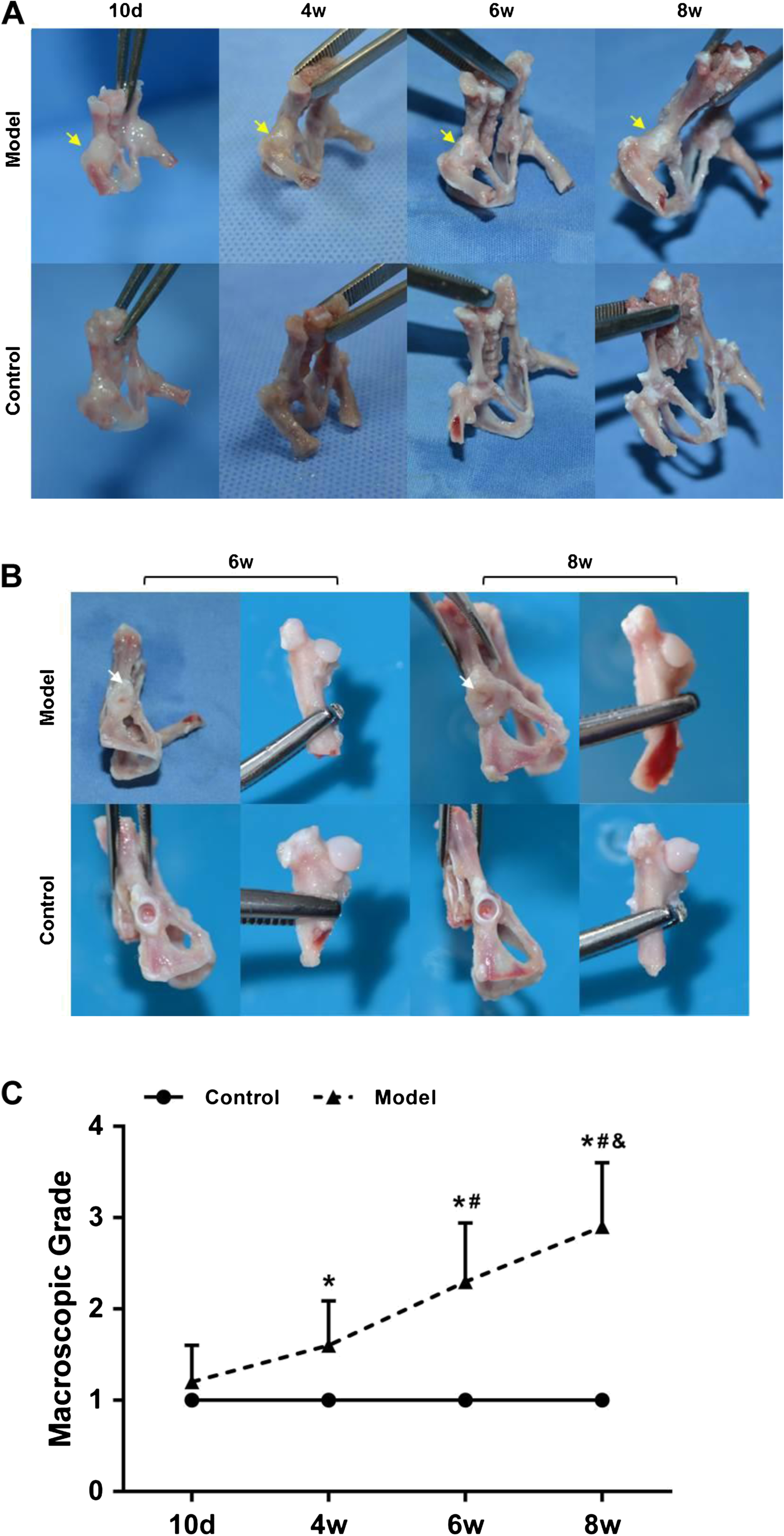

Supplement: Supplementary file 3 — Authors’ original file for figure 1 [file 12891_2014_2374_MOESM3_ESM.tif]

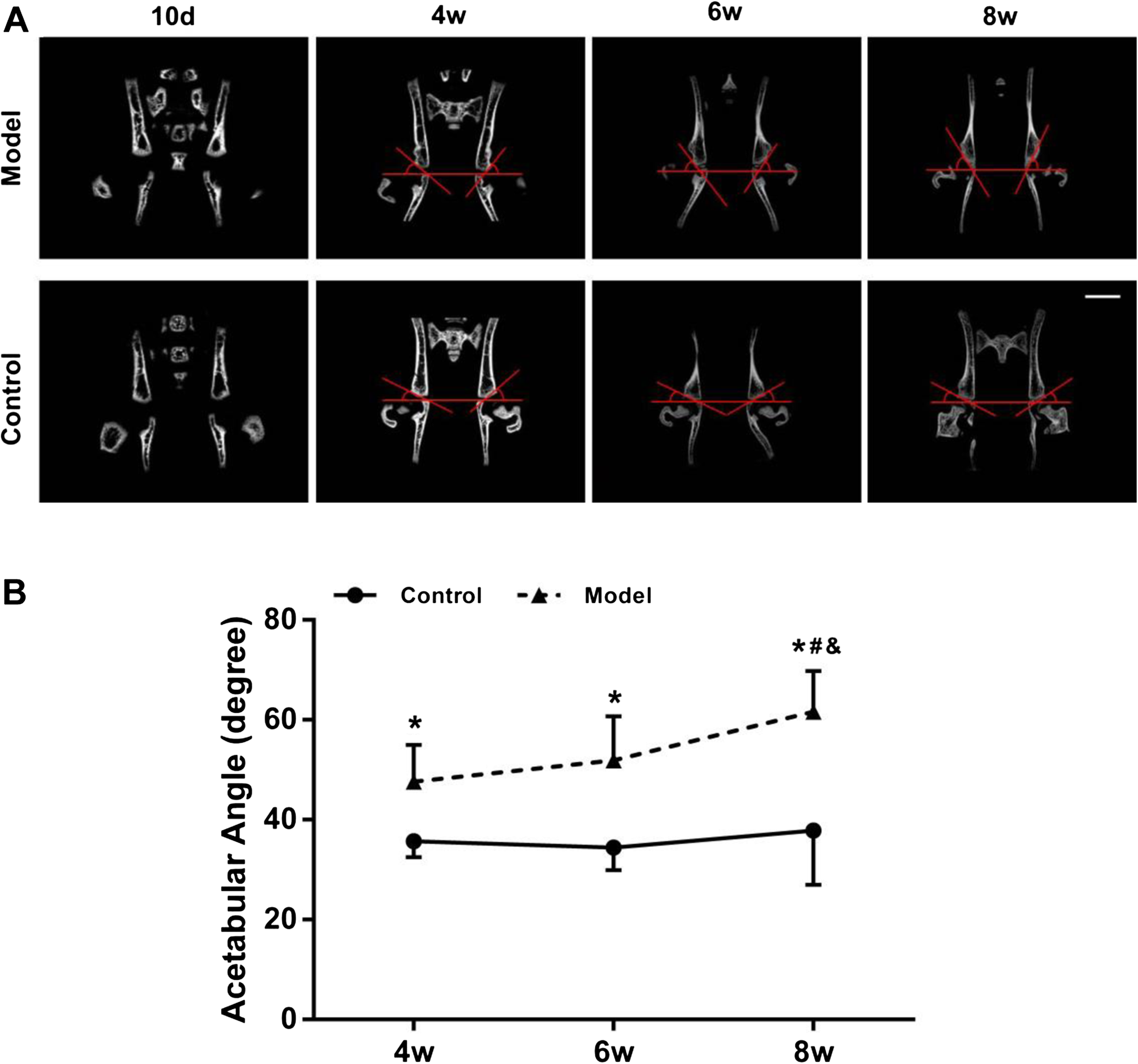

Supplement: Supplementary file 4 — Authors’ original file for figure 2 [file 12891_2014_2374_MOESM4_ESM.tif]

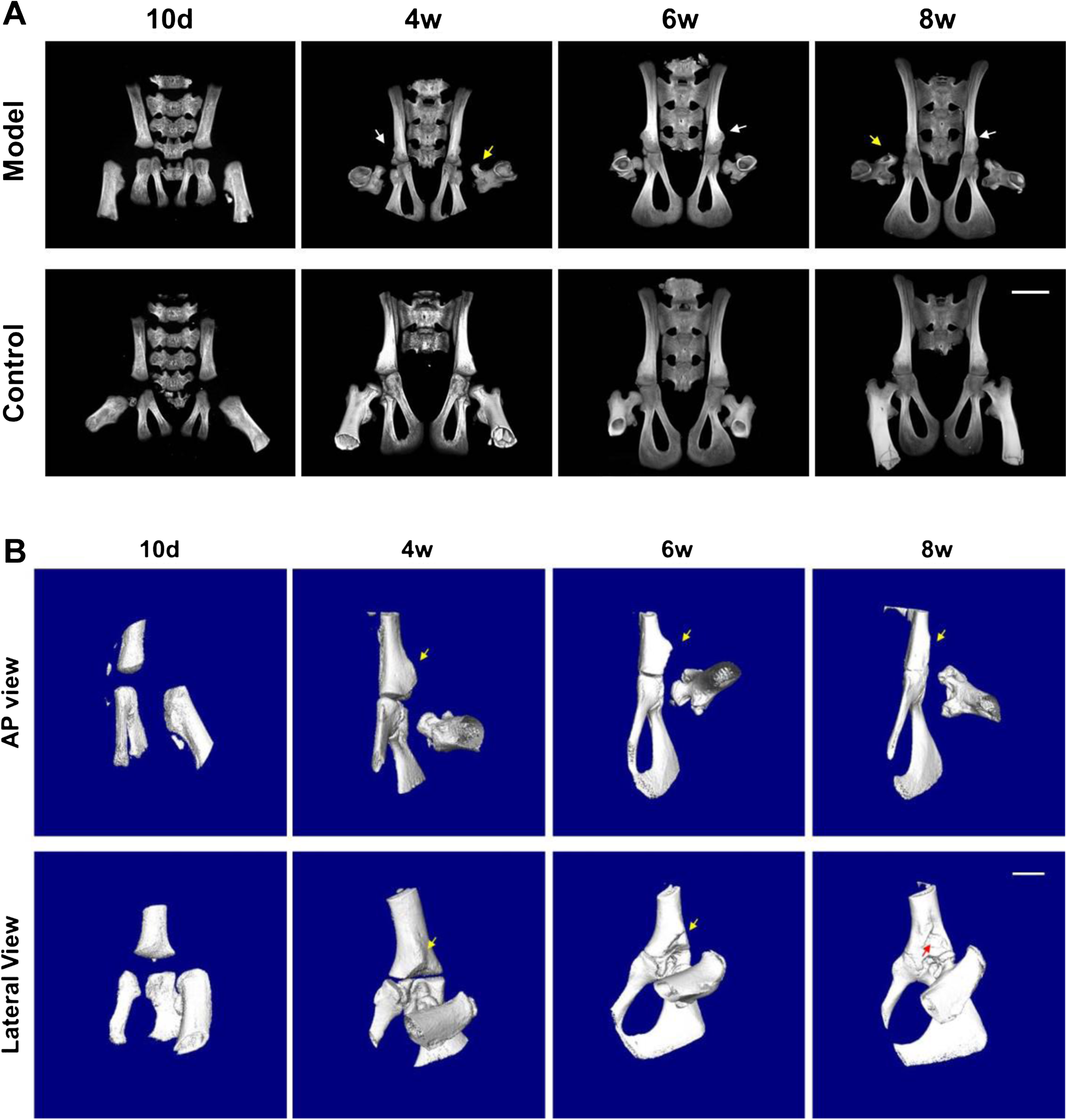

Supplement: Supplementary file 5 — Authors’ original file for figure 3 [file 12891_2014_2374_MOESM5_ESM.tif]

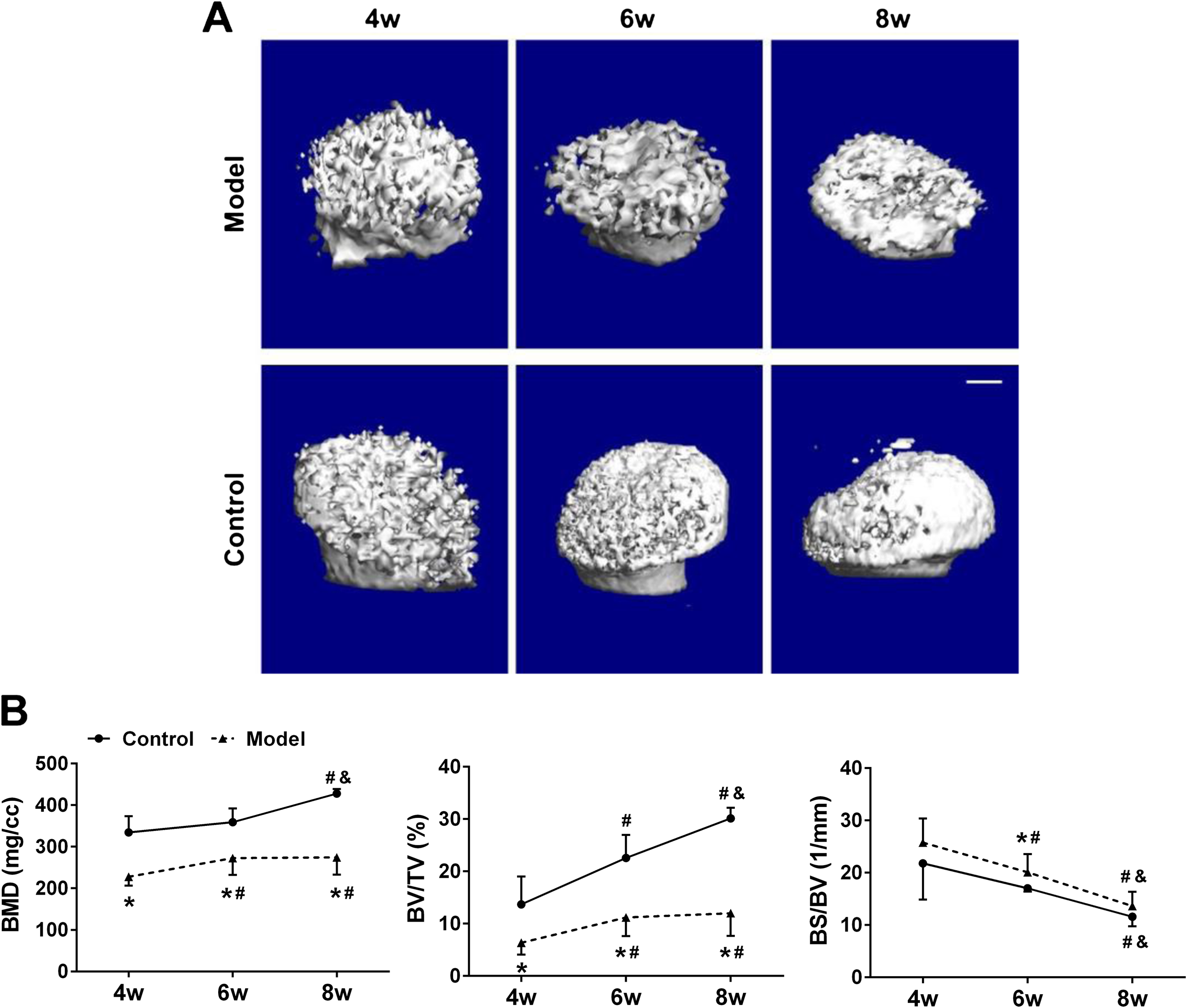

Supplement: Supplementary file 6 — Authors’ original file for figure 4 [file 12891_2014_2374_MOESM6_ESM.tiff]

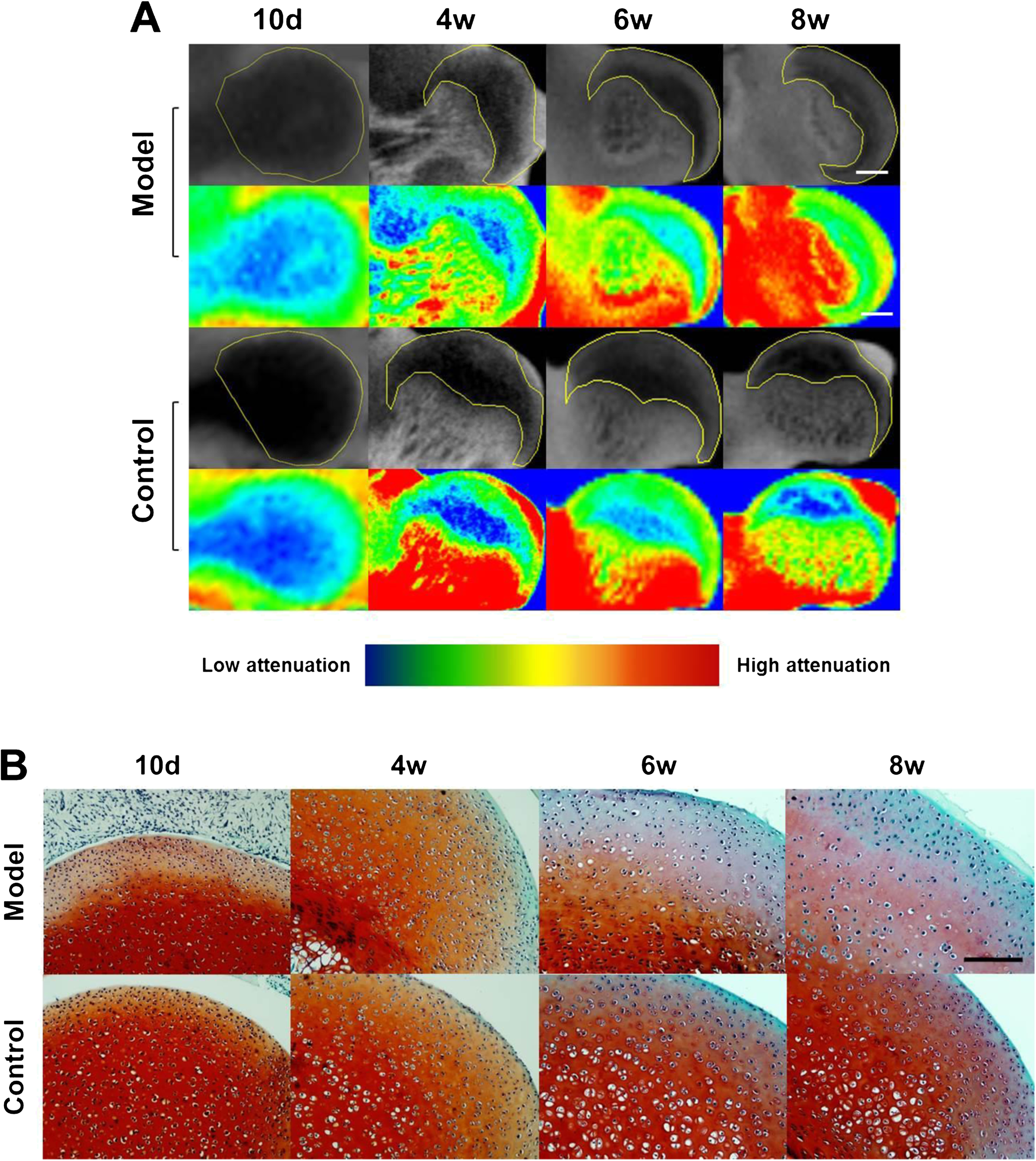

Supplement: Supplementary file 7 — Authors’ original file for figure 5 [file 12891_2014_2374_MOESM7_ESM.tif]

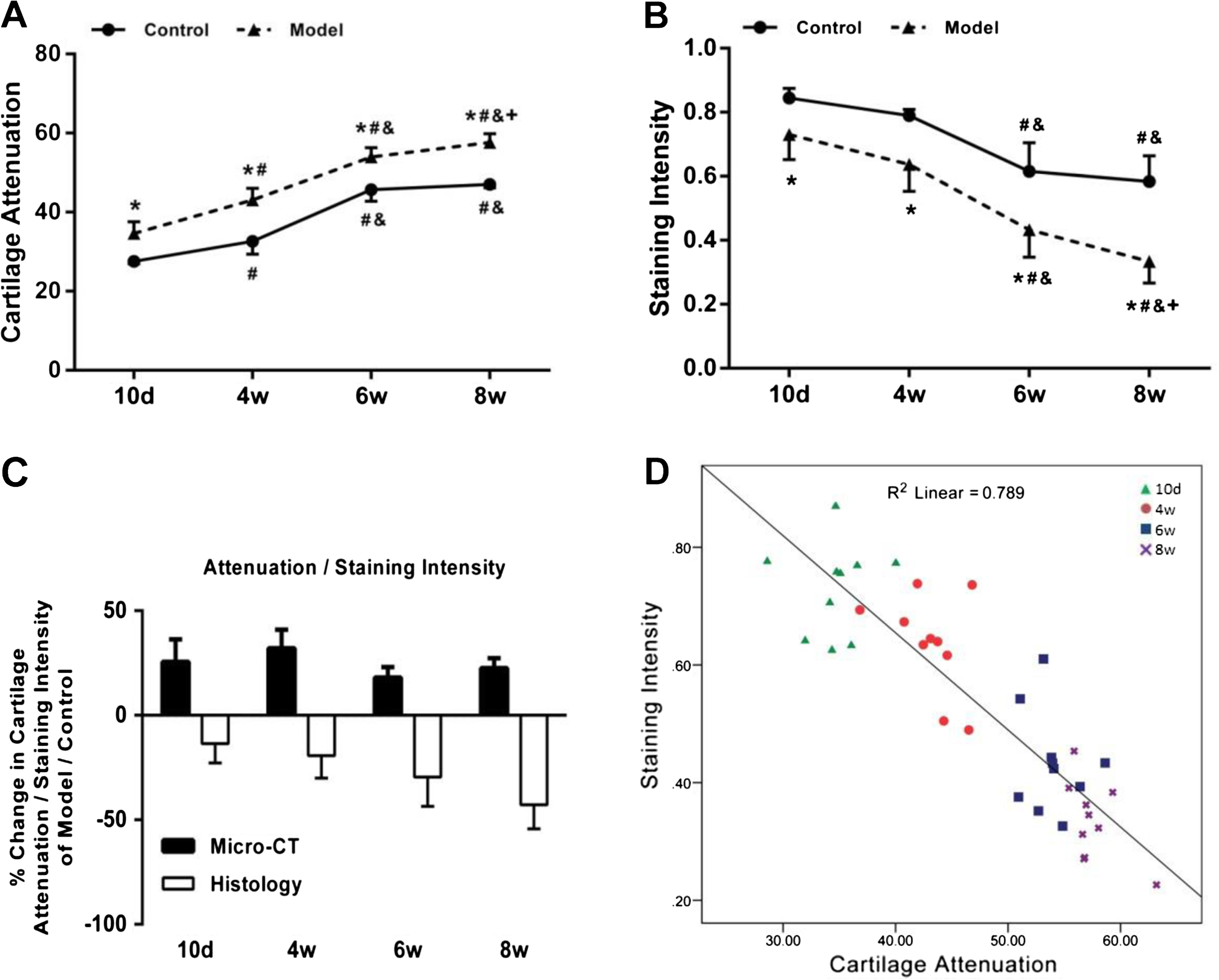

Supplement: Supplementary file 8 — Authors’ original file for figure 6 [file 12891_2014_2374_MOESM8_ESM.tif]
